# Characterisation and *In Silico* Analysis of Interleukin-4 cDNA of Nilgai (*Boselaphus tragocamelus*) and Indian Buffalo (*Bubalus bubalis*)

**DOI:** 10.1155/2013/514145

**Published:** 2013-11-20

**Authors:** M. Saini, T. K. Palai, D. K. Das, K. M. Hatle, P. K. Gupta

**Affiliations:** ^1^Centre for Wildlife, Indian Veterinary Research Institute (IVRI), Izatnagar, Bareilly, Uttar Pradesh 243122, India; ^2^Division of Animal Biochemistry, Indian Veterinary Research Institute, Izatnagar, Uttar Pradesh 243122, India; ^3^Genetic Research Centre, National Institute for Research in Reproductive Health, Mumbai 400012, India; ^4^Department of Cell Biology, Harvard Medical School, Boston, MA 02115, USA; ^5^Division of Veterinary Biotechnology, Indian Veterinary Research Institute, Izatnagar, Uttar Pradesh 243122, India

## Abstract

Interleukin-4 (IL-4) produced from Th2 cells modulates both innate and adaptive immune responses. It is a common belief that wild animals possess better immunity against diseases than domestic and laboratory animals; however, the immune system of wild animals is not fully explored yet. Therefore, a comparative study was designed to explore the wildlife immunity through characterisation of IL-4 cDNA of nilgai, a wild ruminant, and Indian buffalo, a domestic ruminant. Total RNA was extracted from peripheral blood mononuclear cells of nilgai and Indian buffalo and reverse transcribed into cDNA. Respective cDNA was further cloned and sequenced. Sequences were analysed in silico and compared with their homologues available at GenBank. The deduced 135 amino acid protein of nilgai IL-4 is 95.6% similar to that of Indian buffalo. N-linked glycosylation sequence, leader sequence, Cysteine residues in the signal peptide region, and 3′ UTR of IL-4 were found to be conserved across species. Six nonsynonymous nucleotide substitutions were found in Indian buffalo compared to nilgai amino acid sequence. Tertiary structure of this protein in both species was modeled, and it was found that this protein falls under 4-helical cytokines superfamily and short chain cytokine family. Phylogenetic analysis revealed a single cluster of ruminants including both nilgai and Indian buffalo that was placed distinct from other nonruminant mammals.

## 1. Introduction

The discoveries of Interleukin-1 (IL-1) and IL-2 led to a better understanding of the effects of ILs, and till now more than 40 cytokines are discovered with specific functions [1]. Interleukin-4 (IL-4) is one of the extensively studied cytokines which induces specific functions in wide range of immune cells defining its pleotropic character [2]. IL-4 was identified originally as a B cell growth factor-1 in mice [3] and was subsequently shown to modulate other cellular interactions of immune response [4]. It is the primary cytokine which promotes the development of Th2 effector cells and antagonises the activity of interferon gamma (IFN-*γ*) induced development of Th1 cells [5, 6]. Upon activation by IL-4, Th2 cells subsequently produce additional IL-4. These cytokines act synergistically with IL-5 to either activate IgE producing B cells or induce isotype switching and enhance IgE mediated responses in allergy and asthma [7–9]. IL-21 that was discovered recently is homologous to IL-4 in its ability to modulate both innate and adaptive immune responses [10]. Wide diversity of IL-4 activity reported to date suggests that it is a key regulator in humoral and adaptive immunity.

The gene encoding IL-4 is found in chromosomes 11, 5, and 7 in mouse [11], human [12], and cattle [13], respectively. In mouse and human, the gene comprises 4 exons spanning 6 kb and 10 kb, respectively [14, 15]. This cytokine was initially cloned and characterised in mouse and human [16–18]. Further exploration was carried out by characterising it in domestic animals like dog [19], cat [20], camel [21], horse [22], pig [23], and so forth. In addition, IL-4 of some ruminants like cow [24], African buffalo [25], sheep [26], and goat [6] was also previously cloned and identified. In addition, IL-4 has been identified and reported in chimpanzee [27] and bottle-nosed dolphin [28]. Wild animals are presumed to possess stronger immune system as compared to their domestic counterparts. Due to difference in habitat/environment, the immune function of wild animals could be different from that of laboratory bred/domestic animals [29]. A comparison of sequence encoding IL-4 among various wild and domestic species could explain the difference, if any, in structure and function with respect to this cytokine. Indian buffaloes are the centre of dairy industry and are fast replacing indigenous cattle in contribution towards total milk production of India. In India, nilgai is sympatric with one domestic ruminant, that is, Indian buffalo. The present study reports the characterisation of IL-4 of nilgai (*Boselephus tragocamelus*) and Indian buffalo (*Bubalus bubalis*) as a model for the comparison of wild versus domestic ruminants of Bovidae family and their phylogenetic lineage.

## 2. Materials and Methods

### 2.1. Sample Collection and RNA Isolation

Total RNA was isolated from peripheral blood mononuclear cells (PBMs). Blood was obtained aseptically by jugular puncture from nilgai maintained in semicaptivity at Deer Park, Indian Veterinary Research Institute (IVRI), Izatnagar, and Indian buffalo from slaughter house, Bareilly. PBM cells were extracted using Histopaque 1077 (Sigma, USA) density gradient centrifugation following a method previously described [30] and stimulated with Concanavalin A (Con A) at the concentration of 10 *μ*g/mL for 20 h at 37°C in a humidified incubator with 5% CO_2_. Total RNA of both the samples was isolated using Trizol LS reagent (Life Technologies, New York, NY) following the manufacturer's instructions.

### 2.2. cDNA Synthesis and Amplification

Two respective first strands of cDNA were synthesized at 37°C from two RNA samples by using oligo dT primers (Promega, Madison, WI). Nilgai and Indian buffalo IL-4 genes were amplified from their respective cDNA using specific oligonucleotide primers (Forward 5′-TAATGGGTCTCACCTACCAG-3′ and Reverse 5′-TTCAGCTTCAACACTTGGAG-3′) designed based on the sequence of cattle (Accession NM_173921.2). The oligonucleotide primers were designed using OLIGO 4.0 software (USA). The IL-4 specific cDNAs were amplified using sequence specific primers (50 pmol/*μ*L) 1.0 *μ*L each; Template cDNA 1.0 *μ*L; dNTPs (10 mM) 1.0 *μ*L; 10X Taq polymerase buffer 5 *μ*L; 25 mM MgCl_2_ 3 *μ*L; Taq DNA polymerase (MBI Fermentas, 5 U/*μ*L) 1.0 *μ*L; and nuclease free water making final reaction mixture of volume 50 *μ*L. PCR amplification program followed was: 95°C for 5 min, 35 repeated cycles of 1 min denaturation at 94°C, 1 min annealing at 60°C and 1 min extension at 72°C, and one cycle of final extension at 72°C for 10 min. The PCR amplified product was analysed on 1% agarose gel containing ethidium bromide along with DNA molecular weight marker.

### 2.3. cDNA Cloning and Sequencing

The amplified products were purified from the agarose gel using Gel extraction Kit (Qiagen, Germany). Nilgai IL-4 PCR product was cloned into pTZ57R/T vector (MBI Fermentas, MD) and buffalo amplified product using pGEMT-Easy (Promega, Madison, USA) vector following the manufacturers' protocol and further screened by blue white screening. The recombinant plasmids were characterized by restriction enzymes *Not*I, *Pst*I, *Nco*I, and *Eco*RI (MBI Fermentas, MD) and by PCR using gene specific primers predicted from cattle IL-4 sequence.

### 2.4. Sequencing and Analysis

The characterized plasmids were sequenced using T7 and SP6 universal primer using ABI PRISM 377 Version 3.0 DNA sequencer (Applied Biosystem, Foster city, CA). The nucleotide sequences of both insert IL-4 were first BLAST analyzed (http://www.ncbi.nlm.nih.gov/) and further submitted to GenBank. Multiple sequence alignment was carried out with IL-4 gene sequences of nilgai and Indian buffalo with its homologues from other species like cattle (*Bos taurus*) (GenBank Accession no. NM_173921), African buffalo (*Syncerus caffer*) (EU000421), goat (*Capra hircus*) (U34273), sheep (*Ovis aries*) (M96845), pig (*Sus scrofa*) (JF906512), camel (*Camelus dromedarius*) (HM051106), red deer (*Cervus elaphus*) (L07081), giraffe (*Giraffa camelopardalis*) (EU000423), bison (*Bison bonasus*) (EU000422), llama (*Lama glama*) (AB107648), dog (*Canis lupus familiaris*) (NM_001003159), cat (*Felis catus*) (NM_001043339), and bottle-nosed dolphin (*Tursiops truncatus*) (AB020732). 

Amino acid sequences were predicted using DNA Star software (Lasergene). Nucleotide and deduced amino acid sequence were aligned to predict phylograms using Mega 5.1 software [31]. Nilgai and Indian buffalo IL-4 protein structure was predicted using PHYRE2 software (Protein Homology/analog Y Recognition Engine; http://www.sbg.bio.ic.ac.uk/phyre2). The N-glycosylation sites were predicted using HIV sequence database (http://www.hiv.lanl.gov). Leader peptide cleavage site was predicted using SignalP 4.1 server (http://www.cbs.dtu.dk) [32].

## 3. Results 

**Figure 1:**
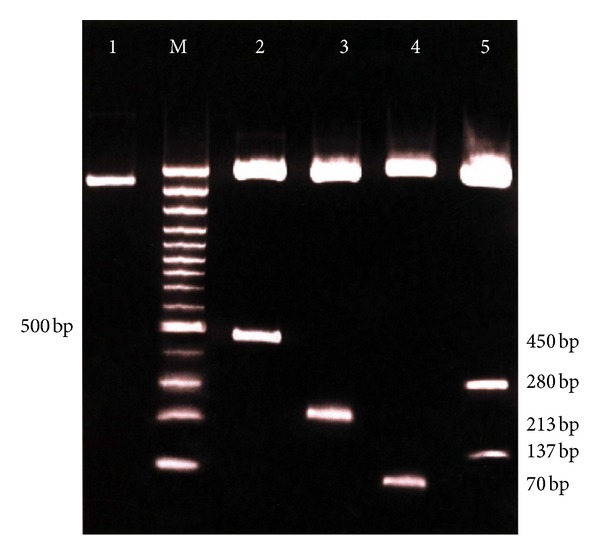
Characterization of recombinant plasmid containing nilgai IL4 by PCR and restriction digestion. L1: linearised recombinant plasmid using BamHI enzyme, M: 100 bp DNA ladder, L2: insert release using *Not*I enzyme, L3: *Pst*I digest, L4: *Nco*I digest, and L5: *Eco*RI digest.

The concentration of RNA was measured using UV spectrometer, and the purity and integrity were checked by analyzing the ratio of optical density (OD) at 260 and 280 nm. The ratios of OD_260_/OD_280_ in total RNA from nilgai and Indian buffalo were found to be 1.83 and 1.85, respectively. Amplification of cDNA through PCR was confirmed through agarose gel electrophoresis which gave a product size 417 bp in both the cases. Purified PCR product of respective species was cloned, and the recombinant plasmid was characterized by restriction analysis (Figure 1, Lanes 2–5) and sequencing. Recombinant plasmid was linearised by digesting with BamH1 (Figure 1, Lane 1); insert was released from vector using *Not*I enzyme (Figure 1, Lane 2). The presence of insert was confirmed by digesting recombinant plasmid with *Pst*I which yielded a single band of around 200 bp (Figure 1, Lane 3). Digestion with* Nco*I yielded product of approximately 70 bp (Figure 1, Lane 4), and *Eco*RI yielded two fragments around 134 and 270 bp (Figure 1, Lane 5). Since the results of characterization of buffalo and nilgai plasmids in agarose gel electrophoresis were identical, the results of nilgai IL-4 are shown in Figure 1. 

The sequences encoding nilgai and Indian buffalo IL-4 cDNA were assigned GenBank accession numbers AY939910 and AY293620, respectively. The full length cDNA of nilgai and Indian buffalo IL-4 contained open reading frame (ORF) of size 408 bp each encoding a protein of 135 amino acids. The molecular weight and isoelectric point of protein were predicted to be 15.039 and 8.785 kDa, respectively, in case of nilgai; however, in case of Indian buffalo these values were found to be 15.158 and 8.940 kDa, respectively.

**Figure 2:**
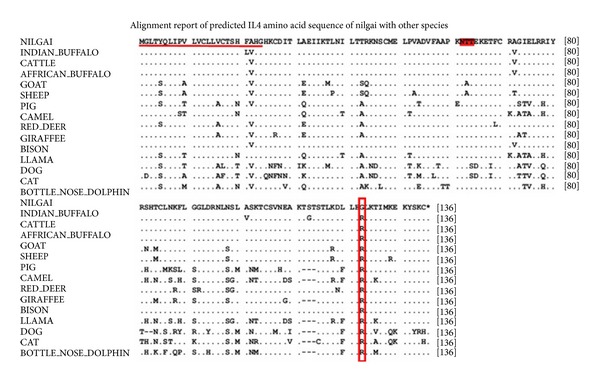
Alignment of predicted amino acid sequence of nilgai IL4 with different species. Identity to the nilgai sequence is indicated by a dot and differences by the corresponding one-letter symbol of the amino acid. Gaps introduced for optimal alignment are indicated by dashes. The conserved leader sequence is underlined; N-glycosylation site is highlighted. One amino acid present in nilgai but replaced in all others is marked with a rectangle.

A comparison of deduced amino acid sequences is provided in Figure 2. Multiple sequence alignment revealed conserved leader sequence of 24 amino acids and a potential N-linked glycosylation site, that is, Asn-Thr-Thr at positions 64–66. Cys (C) residues at positions 13 and 17 in the signal peptide were found to be conserved except in dog and cat for the 17th position. One significant difference is the presence of Gly at position 123 in case of nilgai IL-4 protein, whereas the Arg 123 residue was conserved across other species.

Comparative nonsynonymous nucleotide substitutions leading to change in amino acid at different positions of various species as compared to nilgai are given in Table 1. 

**Figure 3:**
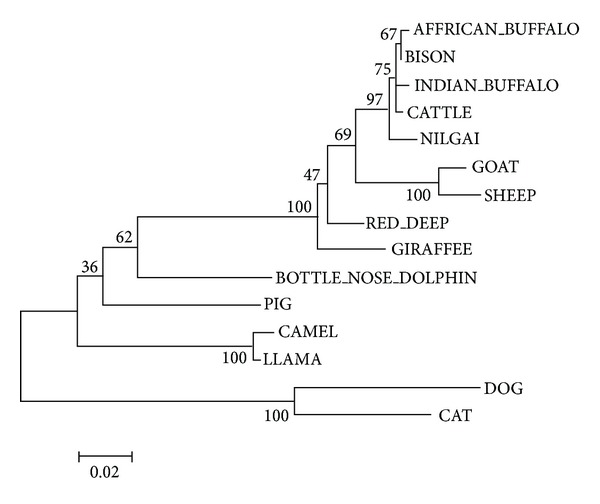
Phylogram illustrating the evolutionary relationship of nilgai and Indian buffalo IL-4 amino acid sequences with other species. The phylogenetic tree was constructed using neighbour-joining analysis. Numbers represent bootstrap values (given as percentages) for a particular node. 1000 replicates were used in bootstrap analysis for good statistical support. The branch lengths are scaled to represent the relative number of substitutions occurring along each branch. The scale bar indicates an evolutionary distance of 0.02 amino acid substitutions per site for a unit branch length.

Phylogenetic analysis based on the nucleotide and predicted amino acid (Figure 3) sequences of IL-4 revealed that the ruminants formed a single cluster indicating their recent divergence from other mammalian species. The phylogenetic tree showed that nilgai IL-4 was more related to cattle, Indian buffalo, African buffalo, and bison than to other species sequence included in the comparison. 

**Figure 4:**
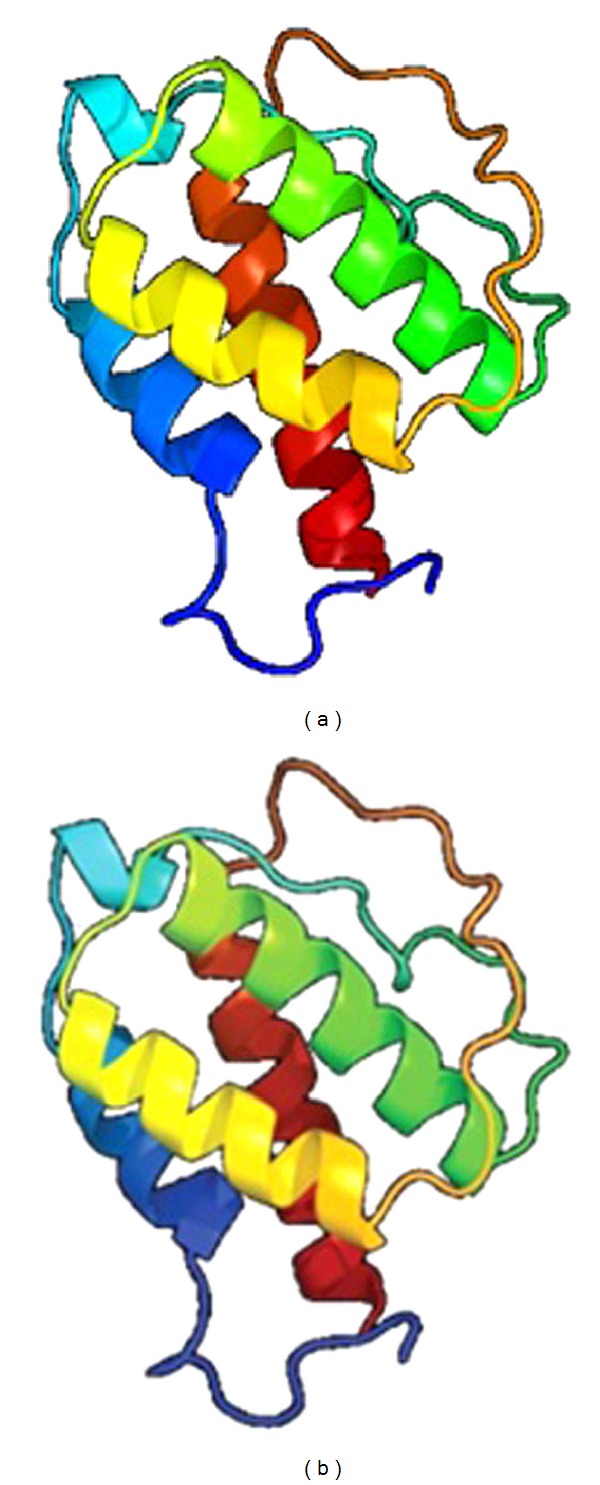
Predicted 3-D structure of (a) nilgai and (b) Indian buffalo IL-4 protein.

From the deduced amino acid sequences, similar tertiary structure was predicted for both Indian buffalo and nilgai IL-4 proteins (Figure 4).

## 4. Discussion

IL-4 is one of the key cytokines in Th2 mediated immune responses, which has been shown to regulate the responses of many other cytokines like IL-1, interferon-gamma, and tumor necrosis factor-alpha. Several reports are there regarding human, murine, and domestic animal IL-4 with scanty reports on wild ruminants. In the present study, nilgai and Indian buffalo IL-4 cDNA was sequenced, and amino acid sequences were predicted for the precursor of the protein in both these species that were further compared with other sequences available in the database. Upon alignment of nilgai IL-4 ORF region sequences with its homologues revealed maximum similarity with cattle (98.3%) followed by Indian buffalo (97.5%), sheep (95.6%), goat (93.9%), camel (85.3%), dolphin (84.3%), pig (81.6%), cat (73.9%), and dog (72.2%). The deduced amino acid sequences showed that nilgai IL-4 is highly similar to cattle with 97.8% match followed by 95.6% with Indian buffalo, 90.4% with goat, and 91.9% with sheep. 

One striking difference is the presence of Gly at position 123 in case of nilgai IL-4 protein instead of conserved Arg 123 residue across other species. At this position, the arginine which is a polar amino acid changed to a nonpolar neutral amino acid glycine. This could lead to minor changes in the folding of protein as evident from the predicted tertiary structure (Figure 4) and cause observed difference in isoelectric point that may influence the activity of this cytokine in both the species.

 Nilgai and Indian buffalo amino acid sequence revealed a leader sequence of 24 amino acids, and their mature peptides are predicted to be of molecular weight 12.44 kDa and 12.56 kDa, respectively. Similar finding was earlier reported in cattle [24].

A potential N-linked glycosylation sequence Asn-Thr-Thr (positions 64, 65, and 66) is found to be conserved in all species. Similar findings were also reported in cattle [24] and in human IL-4 [18]. In addition to earlier reported glycosylation site additional N-glycosylation sites were identified upon *in silico* analysis (Table 1). But whether glycosylations occur in all these sites is not yet to be established. 

Sequence analysis also revealed that the Cys (C) residues at positions 13 and 17 in the signal peptide were found conserved in all the species except in dog and cat for 17th position. Similar result was reported on comparison of ovine and bovine IL-4 sequence [33]. N-linked glycosylation sites and Cys residues were found to be located in the same position in all species. This observation suggests that this region is highly conserved in all the species and may play an important role in determining tertiary structure and functional integrity of the cytokine.

 It was observed that 3′ UTR of IL-4 gene contains A+T rich stretches which include both tandem repeats of TAAT or ATTTA and also the polyadenylation signal sequence. Similar observations were earlier reported in cattle and human [18, 24]. 

Findings of phylogram that ruminants form a cluster, and nilgai IL-4 is evolutionarily closer to buffalo and cattle than other mammals studied, were also corroborated previously on different cytokines of nilgai, that is, in IL-2 [34] and IL-18 [35].

A comparative analysis on nonsynonymous nucleotide substitutions leading to change in amino acid at different positions of various species as compared to nilgai is given in Table 1. In spite of six variations in predicted amino acid sequences, the tertiary structure predicted for both Indian buffalo and nilgai IL-4 proteins was nearly the same (Figure 4). It is evident that both these modeled proteins fall under 4-helical cytokines superfamily and short-chain cytokine family. Since 81% of the amino acid sequences submitted have been modeled with 100% confidence by the single highest scoring template, few alterations in amino acid between two species did not result in change in the predicted structure of the protein.

## 5. Conclusion

This comparison of nilgai and Indian buffalo IL-4 precursors will be useful to correlate the molecular aspect of immunity in wild and domestic ruminants.

## Figures and Tables

**Table 1:** Possible N-glycosylation sites and amino acid substitutions in various species with respect to nilgai IL4 protein.

Species	Possible N-glycosylation sites in IL4		Nonsynonymous nucleotide substitution leading to amino acid change with position with respect to nilgai IL4
	Total number	Position (s)	
Nilgai	01	62	Taken as standard for comparison
Cattle	01	62	A22V, A72V, G123R
African buffalo	01	62	A22V, A72V, G123R
Indian buffalo	01	62	F21L, A22V, A72V, A101V, S113G, G123R
Goat	02	62 and 96	Y5S, V10A, A22V, A32E, T37M, T43S, R44Q, T63A, S82N, T84M, N98S, K118R, G123R
Sheep	02	62 and 96	Y5S, V10A, A22V, A32E, L38P, T43S, R44Q, V53A, T63A, A72T, T84M, N98S, K118R, G123R, K129R
Pig	03	62, 96 and 102	Y5S, V10T, V16A, H20N, A22V, A32Q, T43A, A54T, K61E, G73S, I74T, E75V, R78H, S82H, L96M, N97K, K98S, F99L, G91S, N98S, L100M, S102N, K103M, N108H, L120F, G123R
Red deer	02	62 and 96	Y5S, V16A, A22V, A32E, T43A, F69L, K98R, G91S, G92R, N108S, S109G, D119N, G123R
Camel	04	62, 96, 102 and 108	P9S, V10T, H20N, A32Q, I40T, P52T, R71K, G73A, I74T, E75A, R78H, S82H, T84N, N87S, F89H, G90S, N98S, S99G, S102N, K103T, E109D, A110S, K118R, L120F, G123R, T126K
Giraffe	02	62 and 96	V16A, A22V, C27R, A32E, T43A, G73A, I74T, T84M, K98R, G91S, N98S, E109G, G123R
Bison	01	62	A22V, A72V, G123R
Llama	04	62, 96, 102 and 108	Y5S, V10T, H20N, A32Q, I40T, T43A, P52T, R71K, G73A, I74T, E75A, R78H, S82H, T84N, N87S, F89H, G91S, N98S, S99G, S102N, K103T, E109D, A110S, K118R, L120F, G123R, T126K
Cat	06	28, 45, 62, 84, 96 and 102	G2D, Y5S, V10A, V16A, C17F, H20T, A22V, H25Q, K26N, C27F, D28N, I29N, A32K, T43A, K45N, N46D, P52T, A54M, F57L, T64S, E65D, T68I, G73T, I74T, E75V, R78Q, R81T, T84N, L86S, N87T, G91K, S92H, N98S, L100M, S102N, K101R, A110V, S115C, L120F, G123R, T126A, K129Q, E130K, C135H
Dog	06	28, 45, 62, 83, 95 and 101	Y5S, V10A, V16A, C17L, H20T, A22V, K26N, C27F, D28N, L31I, A32K, T37M, T43A, K45N, N46D, P52T, A54K, A58T, T64S, E65D, T68I, G73A, I74T, E75V, R78Q, R81T, T84N, L86S, K88R, F89Y, G91R, D94Y, N98S, L100M, S102N, V107M, A110I, L120F, G123R, T126V, K129Q, E130K, S133Y, K134R, C135H
Bottle-nosed dolphin	03	62, 96 and 102	Y5S, V10M, V16A, H20N, A22V, I29V, A32Q, T43A, R44K, S47L, A54E, A59T, P60T, I74T, E75V, R78H, S82H, T84K, L86F, K98Q, F99P, G101S, D104H, N108S, L110M, S112N, K113M, L120F, G123R, T126M
